# Dynamic sustainable productivity growth of Dutch dairy farming

**DOI:** 10.1371/journal.pone.0264410

**Published:** 2022-02-25

**Authors:** Liyun Zhu, Alfons Oude Lansink

**Affiliations:** 1 Hebei Agricultural University, Hebei, China; 2 Wageningen University, Wageningen, The Netherlands; Universidad Nacional Autonoma de Nicaragua Leon, NICARAGUA

## Abstract

The economic, environmental and social sustainability of Dutch dairy farms have attracted increasing societal concern in the past decades. In this paper, we propose a recently developed dynamic Luenberger indicator based on the by-production model to measure dynamic productivity growth in the economic, environmental and social dimensions of sustainability of Dutch dairy farms. Subsequently, we investigate the statistical associations between productivity growth and socio-economic factors using the OLS bootstrap regression model. We find that dairy farms have suffered a decline in dynamic sustainable productivity growth, especially in the environmental dimension where it is more pronounced than in the economic and social dimensions. Furthermore, we find that both technical and scale inefficiency change contribute to the decline of environmental productivity growth. Specialization and government support are associated with a higher economic and environmental sustainability productivity growth, and with, a decreased growth of social sustainable productivity. We found no significant association between the age of the oldest entrepreneur, financial structure, farm size or cost of advisory service and dynamic productivity growth in the three sustainability dimensions. The results provide insights into potential pathways towards improving the three pillars of sustainability.

## 1. Introduction

The concept of sustainable agricultural production has become key to policy recommendations in the European Union and elsewhere in the past decades. Montabon et al. [1] proposed that environmental and social issues are increasingly prioritized over economic issues. Dairy farms that use more fertilizer inputs, may put pressure on the environment through air, water and soil emissions [2]. For instance, the nitrogen surplus on many dairy farms results in leaching of soil nitrate to groundwater and loss of nitrous oxide [3]; also, P_2_O_5_ surpluses above 5Kg/ha/year increase the risk of P losses to water [4]. Currently, the Netherlands has the highest N surplus per hectare of agricultural land in the EU, and Dutch dairy cows have the highest per cow emission of N in the EU [5]. From the societal perspective, animal welfare and animal health in dairy farming have been the subject of increasing societal concern [6]. Animal welfare is more broadly seen as one of the factors that determines the sustainability of animal production systems in Europe [7, 8]. Milk yield is associated with lower fertility and higher incidence of production diseases such as mastitis and laminitis, indicating pursuing higher yields comes at the cost of lower welfare of dairy cows in the long term [9]. National governments and stakeholders within the supply chain have reacted to these increasing societal concerns by introducing environmental regulations on emissions and the use of animal manure, and by introducing animal welfare concepts.

The relevant economic literature on sustainability assessment of dairy farms can be classified into two major streams. The first stream focuses on a single dimension of sustainability beyond economic sustainability. The majority of these studies evaluated economic performance [10, 11], and environmental sustainability [12–17]. Only a few studies investigated the social impacts of dairy farms, mainly because of methodological and data shortcomings [18]. Although the welfare assessment of dairy calves [19, 20] as well as the relationship between animal welfare and technical efficiency of dairy farms [21, 22] have been studied, only few studies take animal welfare into account as an input or output indicator of the production process when examining dairy farm performance [23, 24]. The second stream of literature includes integrated sustainability assessments of dairy farms, incorporating economic, environmental and social factors through the MOTIFS approach, sustainability scorecard or sustainability index (SI) [25–28]. (However, the results of these studies rely on stakeholders’ subjective weights of sustainability indicators.

This study proposes the use of Data Envelopment Analysis (DEA) in assessing the sustainability improvements on Dutch dairy farms. DEA has two main advantages that make it a useful method for evaluating sustainability. The first is that it does not require an ex-ante assumption about the functional specification of the production frontier which defines the best performing farms in the sample and indicates the potential for sustainability improvement. Second, the method allows for combining monetary and non-monetary inputs and outputs, i.e., social and environmental sustainability indicators can be included in the analysis along with economic indicators. Nevertheless, comprehensive sustainability assessments incorporating the three sustainability dimensions (economic, social and environmental) are currently lacking [29, 30]. In the existing DEA literature, sustainability has been frequently assessed from the perspective of (in)efficiency for a specific time period. A more recent example of this approach is the seminal work of [31] who introduced the by-production model which can consider two sub-frontiers to characterize the good and bad outputs production process. Dakpo and Oude Lansink [32] extended the by-production model to the dynamic setting, accounting for the adjustment costs associated with investments in new capital assets [33–38]. Studies measuring dynamic productivity growth in each of the three pillars of sustainability are missing to date. Such information could provide guidance for future policy interventions that seek to enhance farm performance and sustainable development. Our review of the relevant literature shows that previous studies on sustainability evaluation of dairy farms have typically addressed two out of the three sustainability dimensions. There are, to the best of our knowledge, no studies that have analyzed the productivity growth of the three dimensions of sustainability, neither in a static context, nor in a dynamic context.

In the light of the foregoing, the objective of this paper is to measure dynamic sustainable productivity growth in the three sustainability dimensions and to identify the main farm and farmer characteristics that drive dynamic sustainable productivity growth. The empirical application focuses on panel data of Dutch dairy farms over the period 2015–2018. This paper contributes to the literature in two ways. First, we develop a new framework of the dynamic Luenberger indicator combined with the by-production model, which can estimate productivity growth of the economic, environmental and social sustainability dimensions simultaneously. Second, the proposed model accounts for sluggish adjustment of quasi-fixed factors. The main research questions addressed in this paper are: (1) what was the dynamic sustainable productivity growth of Dutch dairy farms in the period 2015–2018? (2) What were the main drivers of dynamic sustainable productivity growth on Dutch dairy farms in the period 2015–2018?

The remainder of this paper is organized as follows: the next section outlines the methodology for computing dynamic Luenberger sustainable productivity growth and analyzing the associated relationship between productivity growth and socio-economic farm-and farmer characteristics. Section 3 describes the dataset, the main inputs and outputs used for the dynamic sustainable productivity growth evaluation, as well as the farm- and farmer characteristic influencing the dynamic sustainable productivity growth. Section 4 presents and discusses the empirical results, and the final section offers concluding remarks.

## 2. Method

### 2.1 The Luenberger indicator of dynamic sustainable productivity growth

The model in this paper is inspired by the enhanced Russell-based directional distance measure for assessing the output-specific and input-specific dynamic productivity change proposed by [38]. In order to assess the economic, environmental and social sustainability productivity growth, this paper models the dairy farm’s production technology as the intersection of three sub-technologies: the first and second technology generate the intended economic and social outputs, respectively, whereas the third technology generates the unintended environmental outputs.

Suppose we have a data series in time *t*, consisting of inputs and outputs. The inputs are given by *v*^*t*^, a vector of variable inputs which represents the pollution-generating inputs, *k*^*t*^, a vector of quasi-fixed inputs which are subject to sluggish adjustment through investments *i*^*t*^. *f*^*t*^ is a vector of fixed inputs which represent the nonpollution-generating inputs. The outputs are given by $y_{e}^{t}$, a vector of intended outputs reflecting the economic indicators, $y_{s}^{t}$, a vector of intended outputs reflecting the social indicators and $y_{b}^{t}$, the vector of unintended environmental outputs. The representation of pollution-generating technology in our study is based on the by-production model [31, 39]. The production technology *ψ*^*t*^ is given by the intersection of three sub-technologies, one for the economic good outputs $\psi_{e}^{t}$, the second for the social good outputs $\psi_{s}^{t}$, and the third one for the (bad) environmental outputs $\psi_{b}^{t}$. The production technology *ψ*^*t*^ is represented by $\psi^{t}=\psi_{e}^{t}\cap \psi_{s}^{t}\cap \psi_{b}^{t}$.

Given $\psi_{e}^{t}$ satisfies the free disposability of inputs and intended outputs, and assuming variable returns to scale (VRS), the economical good output production sub-technology in time *t* is defined under a piecewise linear technology as:

$$\begin{array}{l} \psi_{e}^{t}=\{(v^{t},f^{t},k^{t},i^{t},y_{e}^{t},y_{s}^{t},y_{b}^{t}):\\ \sum_{n=1}^{N}\mu_{n}y_{en}^{t}\geq y_{e0}^{t},\\ \sum_{n=1}^{N}\mu_{n}v_{n}^{t}\leq v_{0}^{t},\\ \sum_{n=1}^{N}\mu_{n}f_{n}^{t}\leq f_{0}^{t},\\ \sum_{n=1}^{N}\mu_{n}(i_{n}^{t}-\delta k_{n}^{t})\geq i_{0}^{t}-\delta k_{0}^{t},\\ \sum_{n=1}^{N}\mu_{n}=1,\mu_{n}\geq 0,n=1,\ldots ,N\} \end{array}$$ (1)

Given $\psi_{s}^{t}$ satisfies the free disposability of inputs and intended outputs, and assuming VRS, the social good output production sub-technology in time *t* is defined under a piecewise linear technology as:

$$\begin{array}{l} \psi_{s}^{t}=\{(v^{t},f^{t},k^{t},i^{t},y_{e}^{t},y_{s}^{t},y_{b}^{t}):\\ \sum_{n=1}^{N}\lambda_{n}y_{sn}^{t}\geq y_{s0}^{t},\\ \sum_{n=1}^{N}\lambda_{n}v_{n}^{t}\leq v_{0}^{t},\\ \sum_{n=1}^{N}\lambda_{n}f_{n}^{t}\leq f_{0}^{t},\\ \sum_{n=1}^{N}\lambda_{n}(i_{n}^{t}-\delta k_{n}^{t})\geq i_{0}^{t}-\delta k_{0}^{t},\\ \sum_{n=1}^{N}\lambda_{n}=1,\lambda_{n}\geq 0,n=1,\ldots ,N\} \end{array}$$ (2)

Finally, given $\psi_{b}^{t}$ satisfies the costly disposability of pollution and inputs that cause pollution, and assuming VRS, the environmental bad output production sub-technology in time *t* is defined under a piecewise linear technology as:

$$\begin{array}{l} \psi_{b}^{t}=\{(v^{t},f^{t},k^{t},i^{t},y_{e}^{t},y_{s}^{t},y_{b}^{t}):\\ \sum_{n=1}^{N}\xi_{n}y_{bn}^{t}\leq y_{b0}^{t},\\ \sum_{n=1}^{N}\xi_{n}v_{n}^{t}\geq v_{0}^{t},\\ \sum_{n=1}^{N}\xi_{n}(i_{n}^{t}-\delta k_{n}^{t})\leq i_{0}^{t}-\delta k_{0}^{t},\\ \sum_{n=1}^{N}\xi_{n}=1,\xi_{n}\geq 0,n=1,\ldots ,N\} \end{array}$$ (3)

In total, four DEA models need to be solved to compute the dynamic directional distance functions for two consecutive years *t* and *t*+1:

- two single period Linear Programming models, i.e. one for time *t* (Eq 4) and the second for time *t*+1 (Eq 7)
- two cross-period Linear Programming models, i.e. one for a farm at time *t*+1 in relation to the technology at time *t* (Eq 6), and the second for a farm at time *t* in relation to the technology at time *t* + 1 (Eq 5).

The four models are:

$$\begin{array}{l} {\vec{D}}^{t}(v^{t},f^{t},k^{t},i^{t},y^{t},;{\vec{g}}_{v}^{t},{\vec{g}}_{i}^{t},{\vec{g}}_{ye}^{t},{\vec{g}}_{ys}^{t},{\vec{g}}_{yb}^{t}|CRS)=\underset{\beta ,\mu ,\lambda ,\xi }{\max}\frac{1}{N_{\vec{g}}}(\beta_{e}^{1}+\beta_{s}^{1}+\beta_{b}^{1}+\beta_{v}^{1}+\beta_{i}^{1})\\ s.t.\left\{ \begin{array}{l} \sum_{n=1}^{N}\mu_{n}^{1}y_{ne}^{t}\geq y_{e0}^{t}+\beta_{e}^{1}{\vec{g}}_{ye}^{t}\\ \sum_{n=1}^{N}\mu_{n}^{1}v_{n}^{t}\leq v_{0}^{t}-\beta_{v}^{1}{\vec{g}}_{v}^{t}\\ \sum_{n=1}^{N}\mu_{n}^{1}f_{n}^{t}\leq f_{0}^{t}\\ \sum_{n=1}^{N}\mu_{n}^{1}(i_{n}^{t}-\delta k_{n}^{t})\geq i_{0}^{t}-\delta k_{0}^{t}+\beta_{i}^{1}{\vec{g}}_{i}^{t}\\ \sum_{n=1}^{N}\lambda_{n}^{1}y_{ns}^{t}\geq y_{s0}^{t}+\beta_{s}^{1}{\vec{g}}_{ys}^{t}\\ \sum_{n=1}^{N}\lambda_{n}^{1}v_{n}^{t}\leq v_{n}^{t}-\beta_{v}^{1}{\vec{g}}_{v}^{t}\\ \sum_{n=1}^{N}\lambda_{n}^{1}f_{n}^{t}\leq f_{0}^{t}\\ \sum_{n=1}^{N}\lambda_{n}^{1}(i_{n}^{t}-\delta k_{n}^{t})\geq i_{0}^{t}-\delta k_{0}^{t}+\beta_{i}^{1}{\vec{g}}_{i}^{t}\\ \sum_{n=1}^{N}\xi_{n}^{1}y_{b}^{t}\leq b_{0}^{t}-\beta_{b}^{1}{\vec{g}}_{yb}^{t}\\ \sum_{n=1}^{N}\xi_{n}^{1}v_{n}^{t}\geq v_{0}^{t}-\beta_{v}^{1}{\vec{g}}_{v}^{t}\\ \sum_{n=1}^{N}\xi_{n}^{1}f_{n}^{t}\leq f_{0}^{t}\\ \sum_{n=1}^{N}\xi_{n}^{1}(i_{n}^{t}-\delta k_{n}^{t})\leq i_{0}^{t}-\delta k_{0}^{t}+\beta_{i}^{1}{\vec{g}}_{i}^{t}\\ \sum_{n=1}^{N}\mu_{n}^{1}v_{n}^{t}=\sum_{n=1}^{N}\lambda_{n}^{1}v_{n}^{t}=\sum_{n=1}^{N}\xi_{n}^{1}v_{n}^{t}\\ \sum_{n=1}^{N}\mu_{n}^{1}(i_{n}^{t}-\delta k_{n}^{t})=\sum_{n=1}^{N}\lambda_{n}^{1}(i_{n}^{t}-\delta k_{n}^{t})=\sum_{n=1}^{N}\xi_{n}^{1}(i_{n}^{t}-\delta k_{n}^{t}) \end{array}\right. \end{array}$$ (4)

$$\begin{array}{l} {\vec{D}}^{t+1}(v^{t},f^{t},k^{t},i^{t},y^{t},;{\vec{g}}_{v}^{t},{\vec{g}}_{i}^{t},{\vec{g}}_{ye}^{t},{\vec{g}}_{ys}^{t},{\vec{g}}_{yb}^{t}|CRS)=\underset{\beta ,\mu ,\lambda ,\xi }{\max}\frac{1}{N_{\vec{g}}}(\beta_{e}^{2}+\beta_{s}^{2}+\beta_{b}^{2}+\beta_{v}^{2}+\beta_{i}^{2})\\ s.t.\left\{ \begin{array}{l} \sum_{n=1}^{N}\mu_{n}^{2}y_{ne}^{t+1}\geq y_{e0}^{t}+\beta_{e}^{2}{\vec{g}}_{ye}^{t}\\ \sum_{n=1}^{N}\mu_{n}^{2}v_{n}^{t+1}\leq v_{0}^{t}-\beta_{e}^{2}{\vec{g}}_{v}^{t}\\ \sum_{n=1}^{N}\mu_{n}^{2}f_{n}^{t+1}\leq f_{0}^{t}\\ \sum_{n=1}^{N}\mu_{n}^{2}(i_{n}^{t+1}-\delta k_{n}^{t+1})\geq i_{0}^{t}-\delta k_{0}^{t}+\beta_{e}^{2}{\vec{g}}_{i}^{t}\\ \sum_{n=1}^{N}\lambda_{n}^{2}y_{ns}^{t+1}\geq y_{s0}^{t}+\beta_{s}^{2}{\vec{g}}_{ys}^{t}\\ \sum_{n=1}^{N}\lambda_{n}^{2}v_{n}^{t+1}\leq v_{n}^{t}-\beta_{s}^{2}{\vec{g}}_{v}^{t}\\ \sum_{n=1}^{N}\lambda_{n}^{2}f_{n}^{t+1}\leq f_{0}^{t}\\ \sum_{n=1}^{N}\lambda_{n}^{2}(i_{n}^{t+1}-\delta k_{n}^{t+1})\geq i_{0}^{t}-\delta k_{0}^{t}+\beta_{s}^{2}{\vec{g}}_{i}^{t}\\ \sum_{n=1}^{N}\xi_{n}^{2}y_{b}^{t+1}\leq b_{0}^{t}-\beta_{b}^{2}{\vec{g}}_{yb}^{t}\\ \sum_{n=1}^{N}\xi_{n}^{2}v_{n}^{t+1}\geq v_{0}^{t}-\beta_{b}^{2}{\vec{g}}_{v}^{t}\\ \sum_{n=1}^{N}\xi_{n}^{2}f_{n}^{t+1}\leq f_{0}^{t}\\ \sum_{n=1}^{N}\xi_{n}^{2}(i_{n}^{t+1}-\delta k_{n}^{t+1})\leq i_{0}^{t}-\delta k_{0}^{t}+\beta_{b}^{2}{\vec{g}}_{i}^{t}\\ \sum_{n=1}^{N}\mu_{n}^{2}v_{n}^{t+1}=\sum_{n=1}^{N}\lambda_{n}^{2}v_{n}^{t+1}=\sum_{n=1}^{N}\xi_{n}^{2}v_{n}^{t+1}\\ \sum_{n=1}^{N}\mu_{n}^{2}(i_{n}^{t+1}-\delta k_{n}^{t+1})=\sum_{n=1}^{N}\lambda_{n}^{2}(i_{n}^{t+1}-\delta k_{n}^{t+1})=\sum_{n=1}^{N}\xi_{n}^{2}(i_{n}^{t+1}-\delta k_{n}^{t+1}) \end{array}\right. \end{array}$$ (5)

$$\begin{array}{l} {\vec{D}}^{t}(v^{t+1},f^{t+1},k^{t+1},i^{t+1},y^{t+1},;{\vec{g}}_{v}^{t+1},{\vec{g}}_{i}^{t+1},{\vec{g}}_{ye}^{t+1},{\vec{g}}_{ys}^{t+1},{\vec{g}}_{yb}^{t+1}|CRS)=\underset{\beta ,\mu ,\lambda ,\xi }{\max}\frac{1}{N_{\vec{g}}}(\beta_{e}^{3}+\beta_{s}^{3}+\beta_{b}^{3}+\beta_{v}^{3}+\beta_{i}^{3})\\ s.t.\left\{ \begin{array}{l} \sum_{n=1}^{N}\mu_{n}^{3}y_{ne}^{t}\geq y_{e0}^{t+1}+\beta_{e}^{3}{\vec{g}}_{ye}^{t+1}\\ \sum_{n=1}^{N}\mu_{n}^{3}v_{n}^{t}\leq v_{0}^{t+1}-\beta_{e}^{3}{\vec{g}}_{v}^{t+1}\\ \sum_{n=1}^{N}\mu_{n}^{3}f_{n}^{t}\leq f_{0}^{t+1}\\ \sum_{n=1}^{N}\mu_{n}^{3}(i_{n}^{t}-\delta k_{n}^{t})\geq i_{0}^{t+1}-\delta k_{0}^{t+1}+\beta_{e}^{3}{\vec{g}}_{i}^{t+1}\\ \sum_{n=1}^{N}\lambda_{n}^{3}y_{ns}^{t}\geq y_{s0}^{t+1}+\beta_{s}^{3}{\vec{g}}_{ys}^{t+1}\\ \sum_{n=1}^{N}\lambda_{n}^{3}v_{n}^{t}\leq v_{n}^{t+1}-\beta_{s}^{3}{\vec{g}}_{v}^{t+1}\\ \sum_{n=1}^{N}\lambda_{n}^{3}f_{n}^{t}\leq f_{0}^{t+1}\\ \sum_{n=1}^{N}\lambda_{n}^{3}(i_{n}^{t}-\delta k_{n}^{t})\geq i_{0}^{t+1}-\delta k_{0}^{t+1}+\beta_{s}^{3}{\vec{g}}_{i}^{t+1}\\ \sum_{n=1}^{N}\xi_{n}^{3}y_{b}^{t}\leq b_{0}^{t+1}-\beta_{b}^{3}{\vec{g}}_{yb}^{t+1}\\ \sum_{n=1}^{N}\xi_{n}^{3}v_{n}^{t}\geq v_{0}^{t+1}-\beta_{b}^{3}{\vec{g}}_{v}^{t+1}\\ \sum_{n=1}^{N}\xi_{n}^{3}f_{n}^{t}\leq f_{0}^{t+1}\\ \sum_{n=1}^{N}\xi_{n}^{3}(i_{n}^{t}-\delta k_{n}^{t})\leq i_{0}^{t+1}-\delta k_{0}^{t+1}+\beta_{b}^{3}{\vec{g}}_{i}^{t+1}\\ \sum_{n=1}^{N}\mu_{n}^{3}v_{n}^{t}=\sum_{n=1}^{N}\lambda_{n}^{3}v_{n}^{t}=\sum_{n=1}^{N}\xi_{n}^{3}v_{n}^{t}\\ \sum_{n=1}^{N}\mu_{n}^{3}(i_{n}^{t}-\delta k_{n}^{t})=\sum_{n=1}^{N}\lambda_{n}^{3}(i_{n}^{t}-\delta k_{n}^{t})=\sum_{n=1}^{N}\xi_{n}^{3}(i_{n}^{t}-\delta k_{n}^{t}) \end{array}\right. \end{array}$$ (6)

$$\begin{array}{l} {\vec{D}}^{t+1}(v^{t+1},f^{t+1},k^{t+1},i^{t+1},y^{t+1},;{\vec{g}}_{v}^{t+1},{\vec{g}}_{i}^{t+1},{\vec{g}}_{ye}^{t+1},{\vec{g}}_{ys}^{t+1},{\vec{g}}_{yb}^{t+1}|CRS)=\underset{\beta ,\mu ,\lambda ,\xi }{\max}\frac{1}{N_{\vec{g}}}(\beta_{e}^{4}+\beta_{s}^{4}+\beta_{b}^{4}+\beta_{v}^{4}+\beta_{i}^{4})\\ s.t.\left\{ \begin{array}{l} \sum_{n=1}^{N}\mu_{n}^{4}y_{ne}^{t+1}\geq y_{e0}^{t+1}+\beta_{e}^{4}{\vec{g}}_{ye}^{t+1}\\ \sum_{n=1}^{N}\mu_{n}^{4}v_{n}^{t+1}\leq v_{0}^{t+1}-\beta_{v}^{4}{\vec{g}}_{v}^{t+1}\\ \sum_{n=1}^{N}\mu_{n}^{4}f_{n}^{t+1}\leq f_{0}^{t+1}\\ \sum_{n=1}^{N}\mu_{n}^{4}(i_{n}^{t+1}-\delta k_{n}^{t+1})\geq i_{0}^{t+1}-\delta k_{0}^{t+1}+\beta_{i}^{4}{\vec{g}}_{i}^{t+1}\\ \sum_{n=1}^{N}\lambda_{n}^{4}y_{ns}^{t+1}\geq y_{s0}^{t+1}+\beta_{s}^{4}{\vec{g}}_{ys}^{t+1}\\ \sum_{n=1}^{N}\lambda_{n}^{4}v_{n}^{t+1}\leq v_{n}^{t+1}-\beta_{v}^{4}{\vec{g}}_{v}^{t+1}\\ \sum_{n=1}^{N}\lambda_{n}^{4}f_{n}^{t+1}\leq f_{0}^{t+1}\\ \sum_{n=1}^{N}\lambda_{n}^{4}(i_{n}^{t+1}-\delta k_{n}^{t+1})\geq i_{0}^{t+1}-\delta k_{0}^{t+1}+\beta_{i}^{4}{\vec{g}}_{i}^{t+1}\\ \sum_{n=1}^{N}\xi_{n}^{4}y_{b}^{t+1}\leq b_{0}^{t+1}-\beta_{b}^{4}{\vec{g}}_{yb}^{t+1}\\ \sum_{n=1}^{N}\xi_{n}^{4}v_{n}^{t+1}\geq v_{0}^{t+1}-\beta_{v}^{4}{\vec{g}}_{v}^{t+1}\\ \sum_{n=1}^{N}\xi_{n}^{4}f_{n}^{t+1}\leq f_{0}^{t+1}\\ \sum_{n=1}^{N}\xi_{n}^{4}(i_{n}^{t+1}-\delta k_{n}^{t+1})\leq i_{0}^{t+1}-\delta k_{0}^{t+1}+\beta_{i}^{4}{\vec{g}}_{i}^{t+1}\\ \sum_{n=1}^{N}\mu_{n}^{4}v_{n}^{t+1}=\sum_{n=1}^{N}\lambda_{n}^{4}v_{n}^{t+1}=\sum_{n=1}^{N}\xi_{n}^{4}v_{n}^{t+1}\\ \sum_{n=1}^{N}\mu_{n}^{4}(i_{n}^{t+1}-\delta k_{n}^{t+1})=\sum_{n=1}^{N}\lambda_{n}^{4}(i_{n}^{t+1}-\delta k_{n}^{t+1})=\sum_{n=1}^{N}\xi_{n}^{4}(i_{n}^{t+1}-\delta k_{n}^{t+1}) \end{array}\right. \end{array}$$ (7)

In order to allow for a convenient percentage interpretation of increases in good outputs and decreases in inputs or bad outputs [40] and to avoid infeasibilities [41], the values of the directional vectors are set as the observed values for the variable inputs, the good outputs and the bad outputs, i.e. ${\vec{g}}_{ye}=y_{e0}$, ${\vec{g}}_{ys}=y_{s0}$, ${\vec{g}}_{yb}{=b}_{0}$, ${\vec{g}}_{v}=v_{0}$. Given the variation and frequent occurrence of zero’s in the investment variable, the directional vector is set to 20% of the value of the quasi-fixed assets, i.e. ${\vec{g}}_{i}=0.2\times k_{0}$. Where $N_{\vec{g}}$ represents the number of decision variables in the objective function. We can identify the economic, social and environmental sustainability productivity through $\beta_{e}^{j}.\beta_{s}^{j},\beta_{b}^{j}$.

Following [42], the Luenberger indicator of dynamic productivity change for economic (*L*^EC^), social (*L*^SO^)and environmental (*L*^EN^) sustainability can be denoted as the arithmetic average of productivity change measured by the technology at time *t* + 1 and time *t*. These indicators are as follows:

$$\begin{array}{l} L^{\mathrm{EC}}=\frac{1}{2}(\beta_{e}^{2}-\beta_{e}^{4}+\beta_{e}^{1}-\beta_{e}^{3})\\ L^{\mathrm{SO}}=\frac{1}{2}(\beta_{s}^{2}-\beta_{s}^{4}+\beta_{s}^{1}-\beta_{s}^{3})\\ L^{\mathrm{EN}}=\frac{1}{2}(\beta_{b}^{2}-\beta_{b}^{4}+\beta_{b}^{1}-\beta_{b}^{3}) \end{array}$$ (8)

Following [33], the economic Luenberger indicator of dynamic productivity growth can be decomposed into the contributions of economic dynamic technical change (EC^T^) and dynamic technical inefficiency changes (EC^E^). The economic dynamic technical change represents the shift of dynamic economic production sub-technology between time *t* and time *t* +1. Dynamic technical inefficiency change measures the difference between the value of the dynamic directional distance function under constant return to scale (CRS) at time *t* and time *t* + 1.


$$\begin{array}{l} {\mathrm{EC}}^{T}=\frac{1}{2}(\beta_{e}^{4}‐\beta_{e}^{3}+\beta_{e}^{2}‐\beta_{e}^{1})\\ {\mathrm{EC}}^{E}=\beta_{e}^{1}-\beta_{e}^{4} \end{array}$$ (9)

Building on [42], dynamic technical inefficiency change can be further decomposed into economic dynamic technical inefficiency change under VRS (EC^E/VRS^) and dynamic scale inefficiency change (EC^S^). Dynamic scale inefficiency change measures the difference between the values of the dynamic directional distance functions in CRS and VRS between time *t* and time *t* + 1. These measures are estimated by running the two single-period LP models, corresponding to model (4) after adding the restriction

$$\sum_{n=1}^{N}\mu_{n}^{1}=1,\sum_{n=1}^{N}\lambda_{n}^{1}=1,\sum_{n=1}^{N}\xi_{n}^{1}=1$$ (10)

And model (7) after adding the restriction

$$\sum_{n=1}^{N}\mu_{n}^{4}=1,\sum_{n=1}^{N}\lambda_{n}^{4}=1,\sum_{n=1}^{N}\xi_{n}^{4}=1$$ (11)

The economic dynamic technical inefficiency changes under VRS are then computed as

$${\mathrm{EC}}^{E/\mathrm{VRS}}=\beta_{e}^{1\mathrm{VRS}}-\beta_{e}^{4\mathrm{VRS}}$$ (12)

The dynamic scale inefficiency change can be then computed as:

$${\mathrm{EC}}^{S}=(\beta_{e}^{1}-\beta_{e}^{4})‐(\beta_{e}^{1\mathrm{VRS}}-\beta_{e}^{4\mathrm{VRS}})$$ (13)

Summarizing, the final decomposition of dynamic Luenberger indicator of economic sustainability productivity growth is:

$$L^{\mathrm{EC}}{=\mathrm{EC}}^{T}{+\mathrm{EC}}^{E/\mathrm{VRS}}+{\mathrm{EC}}^{S}$$ (14)

Analogously, the environmental Luenberger indicator can be decomposed into environmental dynamic technical change (EN^T^), environmental dynamic inefficiency change under VRS (EN^E/VRS^) and environmental dynamic scale inefficiency change (EN^S^).


$$\begin{array}{l} L^{\mathrm{EN}}={\mathrm{EN}}^{T}+{\mathrm{EN}}^{E/\mathrm{VRS}}+{\mathrm{EN}}^{S}\\ {\mathrm{EN}}^{T}=\frac{1}{2}(\beta_{b}^{4}‐\beta_{b}^{3}+\beta_{b}^{2}‐\beta_{b}^{1})\\ {\mathrm{EN}}^{E/\mathrm{VRS}}=\beta_{b}^{1\mathrm{VRS}}-\beta_{b}^{4\mathrm{VRS}}\\ {\mathrm{EN}}^{S}=(\beta_{b}^{1}-\beta_{b}^{4})‐(\beta_{b}^{1\mathrm{VRS}}-\beta_{b}^{4\mathrm{VRS}}) \end{array}$$ (15)

The social Luenberger indicator can be decomposed into social dynamic technical change (SO^T^), social dynamic inefficiency change under VRS (SO^E/VRS^) and social dynamic scale inefficiency change (SO^S^).


$$\begin{array}{l} L^{\mathrm{SO}}={\mathrm{SO}}^{T}+{\mathrm{SO}}^{E/\mathrm{VRS}}+{\mathrm{SO}}^{S}\\ {\mathrm{SO}}^{T}=\frac{1}{2}(\beta_{s}^{4}‐\beta_{s}^{3}+\beta_{s}^{2}‐\beta_{s}^{1})\\ {\mathrm{SO}}^{E/\mathrm{VRS}}=\beta_{s}^{1\mathrm{VRS}}-\beta_{s}^{4\mathrm{VRS}}\\ {\mathrm{SO}}^{S}=(\beta_{s}^{1}-\beta_{s}^{4})‐(\beta_{s}^{1\mathrm{VRS}}-\beta_{s}^{4\mathrm{VRS}}) \end{array}$$ (16)

### 2.2 OLS bootstrap regression

The bootstrap approach is used to address the well-known problem of serial correlation among DEA efficiency scores [43]. Since the productivity growth indicators based on DEA are not truncated, the OLS bootstrap regression with heteroskedasticity and autocorrelation robust standard errors is an appropriate approach in our context. Kapelko et al. [33] apply this approach to investigate the impact of food regulation on dynamic productivity growth of the Spanish meat, dairy, and oils and fats industries over the period 1996–2011. In a follow-up study, Kapelko et al. [34] used the same approach to estimate the statistical association between past investment spikes with dynamic productivity growth for oils and fats firms during the same period. The regression model has the following form:

$$y_{it}=\alpha_{i}+\beta \cdot x_{it}+\varepsilon_{it}$$ (17)

Where y_*it*_ indicates the dynamic economic, social or environmental productivity growth for farm *i* in year *t*. *x*_*it*_ is a vector of independent variables that represent farm and farmer characteristics for dairy farm *i* in year *t*, *α*_*i*_ is the constant, *β* is a vector of coefficients to be estimated, *ε*_*it*_ is an error term. Eq (17) allows us to compute bootstrap regression coefficients and bootstrap confidence intervals for the three dynamic sustainable productivity growth measures, with 2000 bootstrap replications.

## 3. Data

Our research focuses on panel data of specialized dairy farms in the Netherlands obtained from the European Commission’s Farm Accountancy Data Network (FADN). Farms were included in the sample in case their revenue from dairy products comprised at least 66% of their total revenue. Taking into account that dynamic Luenberger productivity growth is assessed using pairs of observations from the same farms in two consecutive years, the final data set is unbalanced, consisting of 1096 observations and 775 such pairs during the period 2015–2018.

The DEA model distinguishes two variable inputs *v*^*t*^, two fixed inputs *f*^*t*^, one quasi-fixed input *k*^*t*^, gross investments in quasi-fixed input *i*^*t*^ and three outputs, i.e., economic outputs $y_{e}^{t}$, social outputs $y_{s}^{t}$ and environmental outputs $y_{b}^{t}$. The two fixed inputs are total utilized agricultural area and labor. The former was measured in hectares and the latter was measured in annual working units. The two variable inputs are intermediate consumption and herd size. The intermediate consumption is the aggregation of different operational expenses and structural costs, including the animal feed costs, fertilizers, materials, artificial insemination, energy, seed and veterinary costs. The total expenses were deflated with a Tornqvist price index (base year 2015). Herd size is measured as the number of dairy cows in standardized Livestock Units. The quasi-fixed inputs are measured as the capital stock of buildings and machinery; the total investment includes the annual investments in buildings and machinery, and was expressed in euros of 2015.

The empirical model classifies the three dimensions of sustainability performance as different outputs. The economic performance is quantified here via the good output $y_{e}^{t}$, i.e. total revenue, which includes the total dairy revenue, the arable crops revenue, vegetables revenue, cut flowers revenue and total intensive livestock revenue. The total dairy revenue and the arable crops revenue are the main components. Total revenue was deflated by the Tornqvist price index (base year 2015) of the outputs. The social performance was measured as the number of days dairy cows grazed at least 6 hours and is treated as a good output $y_{s}^{t}$, since grazing may have positive effects on animal welfare [44, 45]. Additionally, Armbrecht et al. [46] concluded that farms with higher daily grazing times (6–10 h and >10 h per day) had better scores with respect to the WQ® principles “good housing” and “good health” compared to farms with lower daily grazing hours or zero grazing. In addition, Wagner et al. [47] found a positive relation between grazing time and animal welfare. With the public’s increasing awareness of animal welfare problems [48, 49], it is closely linked to the social sustainability of animal production. The environmental performance is assessed by considering three bad outputs $y_{b}^{t}$: the greenhouse gas emission, the surplus of nitrogen with farm processing and the surplus of P_2_O_5_ with farm processing. Three GHGs were distinguished, i.e. carbon dioxide (CO_2_), methane (CH_4_) and nitrous oxide (NO_2_), which are aggregated and expressed in kg CO_2_ equivalents. Methane and nitrous oxide are converted to CO_2_ equivalents via the characterization factors published in the IPCC standard (2007), i.e. 1 kg N_2_O is 298 CO_2_ equivalents and 1 kg CH_4_ is 25 CO_2_ equivalents. Table 1 provides the descriptive statistics of the indicators that were used in the empirical analysis.

**Table 1:** Descriptive statistics of the dairy farms (average over the period 2015–2018).

Variables	Dimension	Mean	SD	Min	Max
Land	10 hectares	7.47	4.35	0.95	42.8
labor	full-time equivalent	2.05	1.05	0.7	12.88
Herd size	100 livestock units	1.72	1.06	0.24	9.68
Intermediate consumption	100000 Euro	1.75	1.25	0.07	11.39
Quasi-fixed asset	100000 Euro	6.12	4.61	0.43	44.21
Investments	100000 Euro	1.61	3.22	0	48.71
Total revenue	100000 Euro	5.05	3.67	0.48	50.24
Total GHG emissions	100000 kg of CO_2_-eq	15.99	10.3	1.75	68.73
P_2_O_5_ surplus	1000 kg	13.2	1.83	0.66	24.64
Nitrogen surplus	1000 kg	26.94	9.72	0.3	103.09
Grazing days	10 days	12.87	8.43	0	27
Age oldest entrepreneur	10 years	5.38	0.95	2.7	8.6
Financial structure	ratio	0.65	0.18	−0.07	1
Farm size	1000 Euro	0.52	0.28	0.09	1.88
Specialization	ratio	0.88	0.06	0.67	1.08
Government support	ratio	0.01	0.01	0	0.11
Cost of advisory service	ratio	0.02	0.02	0	0.25

The variables used in the OLS bootstrap regression are presented in the bottom part of Table 1 and are explained in what follows. The relationship between the farm(er)- characteristics and economic productivity are more often present in the literature, whereas the relationship with social and environmental productivity needs further verification in our study.

(1) *Age oldest entrepreneur*. The evidence about the effect of farm households’ age on productivity is ambiguous. In the later stage of their life cycle, farmers will adjust their goal from investing and expansion to improving efficiency, leading to productivity increases with experience. However, older farmers may be unwilling to adopt technological innovations which hinder the productivity growth. Kimura and Sauer [50] found that the age of the farm manager has a positive relationship with productivity growth in the Netherlands, but the opposite is the case in Estonia.

(2) *Financial structure* is described using the debt ratio which is measured as the ratio of debts to assets. It captures the farm’s ability to manage long-term debt and growth over time [51, 52]. It also indicates the resilience of a dairy farm to financial distress. According to the credit evaluation hypothesis, technically efficient firms may easily borrow as they are more likely to repay the debt and borrowers with high credit ratings may obtain more credit [53]. Hence, lack of credit can impede uptake of appropriate technology and limit productivity growth. This could explain a positive relationship between long-term debt and productivity growth [54]. In contrast, [55] proposed that the short run variations in debt servicing ratio are negative and significantly related to productivity growth.

(3) *Farm size* is included via the standard output, which is a size measure used in the FADN based on value added of the outputs. Smaller farms usually face the challenge of attracting professional employees, leading smallholders to employ more family labor per hectare, and that average labor productivity is lower than larger farms [56]. [50] found the farm size was positively associated with the productivity growth of dairy farms in the Netherlands and Estonia. In addition, the principal-agent relationship and asymmetric information between hired labor and managers of large farms may lead to supervision costs which in turn lower productivity growth on large farms [57]. [58] reported that total factor productivity (TFP) decreased with farm size because large farms fail to use their labor resources in full. Factor market transactions costs, coupled with economies of size in farm mechanization, can also lead to a U-shaped pattern of the relationship between TFP and farm size [59]. [60] revealed that farm size was not associated with productivity growth for dairy farms in the southern part of Chile during the period from 2005 to 2010.

(4) *Specialization* is measured as the ratio of the revenue from dairy farming to total revenue. The empirical literature is inconclusive regarding the impact of specialization on productivity growth. On the one hand, specialization may enable farmers to accumulate knowledge in a single production activity, which could positively affect farm performance [61]. Kazukauskas et al. [62] found that increased specialization had positive effects on productivity. On the other hand, more diversified farms may experience economies of scope through cost savings that occur when farms can use the same input for the production of several outputs (see e.g. [36, 63]). Melhim and Shumway [64] reported that economies of scope lead to 27% cost savings when milk, livestock, and crops were jointly produced on the average US dairy farm. In the presence of economies of scope specialization will lead to a lower productivity growth.

(5) *Government support* is included as the ratio of revenue from subsidies to total revenues. The relation between governmental support and a producer’s performance is complex. Subsidies may be positively related with productivity growth by providing a source of financing and credit directly and, hence, enable further investment on innovation. Therefore, some studies reported a positive relation between subsidies and farm productivity growth [62, 65, 66]. On the other hand, subsidies that are coupled to production may decrease producers’ incentives to increase productivity and hinder reallocation of existing resources [67]. Hence, several studies have found a negative relationship with productivity growth [68, 69].

(6) *Cost of advisory service* is included as the ratio of advisory service costs to the total variable cost. By measuring this as a ratio, we avoid capturing size-effects. Through the livestock extension service support, farmers could analyze and improve farm performance, for example through adoption of innovative technologies. Sheng and Chancellor [70] reported that use of contract services to replace self-owned capital may lift the productivity level of small farms. Khan et al. [71] also showed that the provision of reliable livestock extension services positively influences the productivity of dairy farms and ultimately improves dairy farmers’ income.

## 4. Results and discussion

Table 2 shows the arithmetic means of dynamic economic, social and environmental productivity changes and their decomposition into dynamic technical changes, technical inefficiency changes and scale inefficiency changes for the pairs of consecutive years. Please note that the mixed period directional distance functions used to compute the dynamic Luenberger indicator may yield infeasibilities. In our computations, infeasibilities occurred in 2015/2016, 2016/2017 and 2017/2018 for 12.4%, 17.3% and 12.5% of the observations, respectively. Literature suggests two possible solutions for this problem in case of the static Luenberger productivity indicator [34], i.e., omitting the infeasible observations in the computation of averages or assigning to the indices the value zero. In this study, we opted for the first solution, i.e. we excluded infeasibilities in the computation of averages.

**Table 2:** Dynamic sustainable productivity change indicators and their decomposition, 2015–2018.

Dimension	2015/2016	2016/2017	2017/2018	mean
*Economic*				
Productivity change	−0.009	0.010	−0.001	0.000
Technical change	0.010	0.061	−0.045	0.009
Technical inefficiency change (VRS)	−0.025	−0.013	0.011	−0.009
Scale inefficiency change	0.004	−0.031	0.029	0.001
*Social*				
Productivity change	0.007	−0.031	0.014	−0.003
Technical change	−0.016	−0.054	−0.004	−0.025
Technical inefficiency change (VRS)	−0.003	0.001	0.020	0.006
Scale inefficiency change	0.026	0.015	−0.003	0.013
*Environmental*				
Productivity change	−0.015	0.011	−0.028	−0.011
Technical change	0.038	0.040	−0.040	0.013
Technical inefficiency change (VRS)	0.010	−0.002	−0.016	−0.003
Scale inefficiency change	−0.063	−0.257	0.034	−0.095

As for the economic sustainability dimension, the average dynamic Luenberger productivity growth is zero, suggesting no change in performance during the study period. Dynamic technical change and dynamic scale inefficiency change made, on average, positive contributions to dynamic productivity growth, while dynamic technical inefficiency change contributed negatively. The average technical progress is 0.9%, which is line with [50], who show that the productivity of the Dutch dairy farm improved through adoption of new technologies during the period 2001–2012. Skevas et al. [72] also apply the dynamic stochastic frontier model and found that technical progress is the main driver of TFP growth for German dairy farms in the period 2001–2009. The dynamic scale inefficiency increased by 0.1%, suggesting that farms have succeeded in moving the scale of the firm towards constant returns to scale. The dynamic technical inefficiency decreased by 0.9%, which suggest that the farms on average used the existing production technology less efficiently. Our results show some similarity with the study of [33], who also reported a zero value for dynamic Luenberger productivity growth of Spanish dairy farms during the period 1996–2011. However, they found a technical change, a technical inefficiency change and scale inefficiency change of −1.1%, 0.9% and 0.2%, respectively. Nevertheless, our finding points to larger changes than the ones identified by [73], who used the Malmquist index to assess the productivity growth of European dairy farms from 2004 to 2012, and reported that both total productivity change and technological change were −2.3%, and pure efficiency change and scale efficiency change was 0 in the Netherlands. Latruffe et al. [74] also used the static Malmquist index to assess the French dairy farms’ TFP change of −0.7%, with a technological change of 2.6%, a pure technical efficiency change of −3% and a scale efficiency change of −0.2% during the period 2001–2007.

As for the social sustainability dimension, the average dynamic Luenberger productivity growth estimate was approximately −0.3% per year. While the productivity increased in 2015/2016 and 2017/2018, it did not make up for the large decrease in productivity in 2016/2017. Overall, social sustainability productivity worsened during the entire study period, and this was due to a technological deterioration of −2.5% on average. Both the technical inefficiency change and scale inefficiency change made positive contributions to social sustainability productivity growth, with a progress of 0.6% and 1.3% on average, respectively. There are no previous studies that evaluated the productivity change of social output.

Compared with the economic and social sustainability dimension, the average dynamic Luenberger productivity growth of the environmental dimension was the lowest, i.e., −1.1% each year. In contrast to the social dimension, the decrease in environmental sustainability productivity is accompanied by a technological progress of 1.3% on average, whereas, a deterioration in technical inefficiency and scale inefficiency of −0.3% and −9.5%, respectively, occurred. Our environmental technical inefficiency findings are similar to those of [32], who showed that the GHG emissions inefficiency of French suckler cow farms in 1978–1992, 1993–2005 and 2006–2014 was 0.080, 0.056 and 0.067, respectively. The GHG emissions inefficiency change during the entire period 1978–2014 was smaller than 0.

Table 3 presents the corresponding parameter estimation results of economic, social and environmental sustainability productivity change using the OLS bootstrap regression model. We can conclude that specialization is positively and significantly related with economic and environmental productivity change, but negatively and significantly associated with social sustainability productivity change. More specialized dairy farms may have an advantage, ceteris paribus, in the use of land, labor, and fodder; hence, they can simultaneously increase output and decrease the environment load per unit of output. Forgacs [75] reported that the average farm output of specialized farms exceeds that of non-specialized farms and concluded that the former has achieved higher growth in land, labor and total factor productivity. However, excessive stock density and the pursuit of higher milk yields may not take dairy cows welfare into account, therefore leading to a decrease in social sustainability productivity.

**Table 3:** OLS bootstrap regression results of economic, social and environmental sustainability productivity growth and its components.

	Economic productivity	Social productivity	Environmental productivity
Intercept	−0.131***	0.191***	−0.339***
Age oldest entrepreneur	0.001	−0.002	0.002
Financial structure	0.026	0.007	0.011
Farm size	0.007	0.010	−0.011
Specialization	0.117***	−0.221***	0.356***
Government support	0.323*	−0.210	0.789***
Cost of advisory service	−0.038	0.137	−0.193

Notes

***, ** and * indicate about significance at 1%, 5% and 10% significance level, respectively.

The ratio of revenue from subsidies to total revenues is also positively and significantly associated with economic and environmental sustainability productivity change, suggesting that dairy farms that received subsidies had a relatively higher economic and environmental productivity growth. Similar results were reported by [76], who found that subsidies are positively and significantly related with total productivity change for French dairy farms over the period 1995 to 2002. Kleinhanss [77] also found that TFP growth was slightly higher on small German farms with subsidies than on large farms without subsidies. Our results show that government support is not related with social sustainability productivity growth. This suggests that, dairy farmers are more inclined to use government subsidies to purchase automation equipment and expand the scale of production, rather than to focus on improving production systems that enable outdoor grazing. In line with this, Latruffe and Desjeux [78] found that subsidies linked to production were positively and significantly related with productivity change and pure technical efficiency change.

The other farm- and farmer- characteristics, i.e. *age oldest entrepreneur*, *financial structure*, *farm size and cost of advisory service* are not significantly related with economic, social and environmental sustainability productivity growth, indicating these factors are generally not important for dynamic productivity growth.

## 5. Conclusion

This paper introduced a dynamic Luenberger measure of dynamic productivity changes in the economic, social and environmental dimensions, and applied it to a sample of Dutch dairy farms over the period 2015–2018. Our results suggest that over the entire study period, the Dutch dairy farms’ economic sustainability productivity did not change, whereas, dynamic social and environmental sustainability productivity change decreased by -0.3% and -1.1%, respectively. The component analysis of the dynamic sustainable productivity change showed that the decrease in social sustainability productivity was mainly driven by technical regress, whereas the decrease in environmental sustainability productivity was mostly due to a decrease of pure technical and scale inefficiency change, and thus attributable to inefficient management practices and diseconomies of scale. In addition, the results from the bootstrap regression model indicated that *specialization* and *government support* affected the dynamic sustainable productivity.

The results of this paper clearly indicate a decrease in social and environmental productivity change, and identify their main drivers. Furthermore, it provides insights into their association with farm(er)-specific characteristics. Policy-makers and dairy farmers may use the findings of this study to improve the productivity of the economic, social and environmental sustainability dimensions. Firstly, our finding that, social sustainability productivity change is negatively affected by technical regress implies the need for better grazing management systems, which would make outdoor grazing more attractive to farmers. Policy makers could play a role here by enhancing research into more productive outdoor grazing systems. Secondly, our finding that technical inefficiency change and scale inefficiency change are the main drivers of the decrease in environmental sustainability productivity change has two implications: (i) it implies the need for better on farm use of feed inputs and fertilizers, and (ii) adjusting the farm scale can help to reduce the N and P surplus on farms and enhance their environmental performance. Thirdly, both specialization and the ratio of revenue from subsidies to total revenues appear to be negatively related with social sustainability productivity growth, but are positively associated with economic and environmental sustainability productivity growth. Thus, farmers should be made aware of these trade-offs, for example by making use of professional advisors. Policy makers can motivate dairy farmers by coupling the use of subsidies to enhancing animal welfare, e.g. by requiring more outdoor grazing.

Moving to the limitations of this research, we measure animal welfare as the number of days cows are grazing outside. Future research could extend animal welfare to include animal disease prevalence. In addition, other social sustainability indicators could be integrated such as indicators that account for the contribution of farmers to rural livelihood. Future research may also investigate the role of advisory services in enhancing productivity growth in other countries and economic sectors.
